# A Population-Based Study of Unintentional Injury and Premature Death among Non-imprisoned and Imprisoned Youth Offenders

**DOI:** 10.1016/j.jcrimjus.2022.102009

**Published:** 2023-01

**Authors:** Rebecca Siponen, Anneli Andersson, Sofi Oskarsson, Miguel Garcia-Argibay, Amber L. Beckley, Niklas Långström, Seena Fazel, Zheng Chang, Henrik Larsson, Brittany Evans, Catherine Tuvblad

**Affiliations:** 1 School of Law, Psychology and Social work, Örebro University, Örebro, Sweden; 2 School of Medical Sciences, Örebro University, Örebro, Sweden; 3 Stockholm University, Department of Criminology, Stockholm University, Stockholm, Sweden; 4 Centre for Psychiatry Research, Department of Clinical Neuroscience, Karolinska Institute, Stockholm, Sweden; 5 Department of Psychiatry, University of Oxford, Oxford, UK; 6 Department of Medical Epidemiology and Biostatistics, Karolinska Institute, Solna, Sweden

## Abstract

**Abstract Background:**

Youth offenders have a high risk of being injured or dying prematurely. However, few studies have considered the role of imprisonment and potential childhood risk factors for these high rates.

**Aim:**

To examine the risk of unintentional injury and premature death in non-imprisoned and imprisoned youth offenders, and to examine the role of parental criminal convictions and psychiatric disorders and own childhood psychiatric disorders.

**Methods:**

All individuals (N = 1,839,711) born in Sweden between 1978 and 1996 were identified using Swedish population-based registers. The exposure was criminal conviction between ages 15–20 years of age.

**Results:**

Imprisoned youth offenders had the highest risk for unintentional injury (HR = 2.29 [2.19–2.40]) and premature death (HR = 10.76 [9.52–12.16]), followed by nonimprisoned youth offenders, compared to non-convicted youth. All childhood risk factors increased the risk for these outcomes among non-imprisoned youth offenders. Among imprisoned youth offenders, parental criminal convictions and parental psychiatric disorders increased the risk for unintentional injury, and parental psychiatric disorders and own childhood psychiatric disorders increased the risk for premature death.

**Conclusions:**

Our study shows there are robust modifiable childhood risk factors for injury and mortality among youth offenders. However, the importance of them to assess risk may differ between non-imprisoned and imprisoned youth offenders.

## Introduction

Relative to the general population, individuals who commit crime have a higher risk of being seriously injured or dying prematurely (Skinner & Farrington, 2020; Timonen et al., 2003; Zlodre & Fazel, 2012). Previous studies have consistently found an increased risk for injuries and mortality among offenders across different sample types, countries, and offender groups (see Skinner & Farrington, 2020; Zoldre & Fazel, 2012, for review). This risk is particularly true among adolescent and young adult offenders since previous research found that a younger age at first conviction was associated with a higher risk for premature death caused by external causes (Kjelsberg & Laake, 2010; Zlodre & Fazel, 2012). Despite this, most previous studies on injuries and premature death among offenders were conducted using adult samples. Generalizing from adults to youth is problematic for several reasons, including biological, mental, and social differences (Eriksson, 2012; Richards, 2011; United Nations, 1989, 2013; World Health Organization, 2014). Furthermore, youth and adults are generally treated differently by the criminal justice system (Eriksson, 2012; Janson, 2004; Schmidt, Rap, & Liefaard, 2021). Due to these differences, research that focuses specifically on examining injuries and premature death among youth offenders is motivated.

To target interventions at youth offenders at greatest risk of injury or premature death, research needs to examine the role of potential risk factors, such as psychiatric disorders and substance use. Although several studies found that, among youth offenders, psychiatric disorders (e.g., psychosis, personality disorder, and depression) and substance use disorders increased the risk for injuries and premature mortality (Chassin, Piquero, Losoya, Mansion & Schubert, 2013; Laub & Vaillant, 2000; Salias et al., 2006; Stenbacka, Moberg & Jokinen, 2019; Stenbacka & Jansson, 2014), most studies failed to adjust for several important and well-established demographic confounders including socio-economic status (SES), sex, and age. Furthermore, it is well known that criminality (Frisell, Lichtenstein, & Långström, 2011), psychiatric disorders (Pettersson et al., 2019), and premature death (Oyen, Boyd, Poulsen, Wohlfahrt, & Melbye, 2009) aggregate in families. Yet, most studies did not consider the role of family history when examining the association between criminality and injuries or death. A previous population-based study that did adjust for demographic confounders and for familial confounding (i.e., genetic and shared environmental influences) found that among adult offenders released from prison in Sweden, substance use disorder, but no other psychiatric disorders, increased the risk for premature death (Chang, Lichstenstein, Larsson, & Fazel, 2015). Taken together, this suggests that both a person’s own psychiatric disorders and family history of criminal offending and psychiatric disorders may be important to better understand the associations between criminality and unintentional injuries and premature death. These factors need to be further examined in a youth offender sample.

The associations between risk factors in childhood (e.g., psychiatric disorders or family history of criminality), criminal behavior in youth, and the risk for injuries and premature death can be understood from a biopsychosocial perspective. The biopsychosocial perspective suggests that genes and environment work in tandem to create impulsive, antisocial, and criminal tendencies (Raine, 2013). These tendencies could in turn increase both the risk for criminal behavior and for other risky behaviors (e.g., substance misuse, dangerous driving, or fighting; Latvala, Kuja-Halkola, Almqvist, Larsson & Lichtenstein, 2015; Raine, 2015) that increase the risk for being injured or dying prematurely. Researchers have found a wide range of childhood risk factors for criminality, from birth complications (Oskarsson et al., 2022), early temperament (Jolliffe & Farrington, 2019), parental rearing (Fagan & Benedini, 2019), family criminality or psychiatric disorders (Björkenstam, Hjern, Björkenstam, & Kosidou. 2018; Fagan & Benedini, 2019), antisocial peers (Sullivan, Childs & Gann, 2019), school environment (Pepler, 2019), to childhood psychiatric disorders (Beaudry, Yu, Långström & Fazel, 2021; Whiting, Lichtenstein & Fazel, 2021). Some of these risk factors have also been shown to increase the risk for mortality (Brown et al., 2009; van de Weijer, Smallbone & Bouwman, 2018), with additive risks if the individual has also engaged in criminal behavior (e.g., Björkenstam et al., 2008; Fazel & Runeson, 2020; Mok et al., 2016; Salias et al., 2006). Although more research is needed among youth offenders specifically, this indicates that childhood factors could increase the risk for both criminality and mortality separately but are most likely to also have a cumulative effect on mortality risks among youth offenders.

What also needs to be considered when examining risks among youth offenders is that it is not a homogenous group. Research is consistent that youth offenders that commits frequent or more serious crimes have a higher risk for mortality than other offenders (Chassin et al., 2013; Laub & Vaillant, 2000; Nieuwbeerta & Piquero, 2008; Skinner, Farrington & Jolliffie, 2022; Stenbacka & Jansson, 2014; Zane, Welsh & Zimmerman, 2019). Depending on age, offence severity, and partly on treatment needs, youth offenders are given different sanctions in various settings (e.g., residential care or imprisonment vs. non-custodial interventions). In general, youth offenders that commit serious crimes (mainly violent crimes) or are thought to have a high risk to recidivate into other serious offences are sentenced to imprisonment (Eriksson, 2012; SFS 1964:167). These imprisoned youth offenders tend to have an overall high-risk profile for both criminal behavior and adverse outcomes whereas non-imprisoned youth offenders are a more heterogenous group with a larger variety of offences, treatment needs, and risk profiles. Research is conclusive that imprisoned youth offenders have a higher risk for injuries and death compared to the general youth population (Coffey, Veit, Wolfe, Cini, & Patton, 2003; Farrer, Frost, & Hedges, 2013; Hughes et al., 2015; Sailas et al., 2006; Timonen et al., 2003). Remarkably, few studies considered imprisoned *and* non-imprisoned youth offenders separately and findings from these studies have been inconclusive (Aalsma et al., 2016; Kinner et al., 2015). Given the potential differences of risk profiles within these groups, it is necessary to examine the role of childhood risk factors for unintentional injury and premature death *within* these groups separately. This would not only add to research comparing risk for injuries and death *between* these groups (e.g., Aalsma et al., 2016; Kinner et al., 2015), but it would also have implications for our policies on youth crime, crime prevention, health, and social work. Since clinicians and criminal justice professionals work with these youth in different parts of the criminal justice system (e.g., residential facilities vs social services), it would be informative to be able to identify modifiable risk factors that contributes with the highest risk for these outcomes within non-imprisoned and imprisoned youth offenders separately.

In the present study we set out to expand on previous research by examining differences in risk for unintentional injury and premature death in a population-based sample of youth offenders with and without an imprisonment sentence. The aim of the present study was two-fold. First, we aimed to compare the risk for unintentional injury and premature death among non-convicted youth, non-imprisoned youth offenders, and imprisoned youth offenders. Second, we aimed to examine the role of childhood psychiatric disorders, parental criminal convictions, and parental psychiatric disorders in the risk for unintentional injuries and premature death within non-imprisoned and imprisoned youth offenders separately.

## Methods

### Data sources

The present study used a cohort design and data were merged from several Swedish population-based registers via the unique personal identity number (PIN) that every individual in Sweden is assigned at birth or receives upon immigration to Sweden (Ludvigsson, Otterblad-Olausson, Pettersson, & Ekbom, 2009). We linked data from the following registers: the *Total Population Register* (TPR) which contains information on demographic factors (e.g., sex, date and place of birth, civil status) on all Swedish residents born since 1932 and later (Ludvigsson et al., 2016); the *National Crime Register* (NCR) which contains information on all criminal convictions in lower courts in Sweden since 1973; the *National Patient Register* (NPR) which contains complete information on diagnoses based on the Swedish versions of the International Classification of Diseases (ICD), including ICD-8 (1973-1986), ICD-9 (1987-1996), and ICD-10 (since 1997), from inpatient care since 1987 and specialist outpatient care from 2001 (diagnoses from primary care are not included so only more severe cases are covered; Ludvigsson et al., 2011); the *Cause of Death Register* which contains information on the date of death and underlying and contributing causes of death according to the ICD-system (Brooke et al., 2017); the *Multi-Generation Register* which identifies the biological and adoptive parents of every person born 1932 or later and resides in Sweden since 1961; and lastly the *Longitudinal integration database for health insurance and labor market studies* (LISA) which contains yearly assessments of income, employment status, and education levels for all individuals aged 16 years or older since 1990. The linkage of registers has been approved by the Regional Ethical Review Board in Stockholm (2013/862-31/5).

### Study population

We identified all individuals in Sweden who were born between 1978 and 1996 (N=2,590,861). Since the exposure in the present study was criminal convictions and the age for criminal responsibility in Sweden is 15 years of age, we identified all individuals who were alive and residing in Sweden on their 15^th^ birthday using the TPR. We therefore excluded those who had died (*n*=16,063) or emigrated (*n*=38,593) before their 15th birthday.

Immigration during childhood or adolescence could lead to missing information on important childhood risk factors such as childhood psychiatric disorders, a risk factor considered in the present study. Similarly, inability to link individuals to their biological mother or father could lead to missing information on other childhood risk factors such as family history of crime or parental SES. Missing information on childhood risk factors could lead to error in results due to differential information bias, i.e., misclassification of childhood risk factors due to inaccurate measuring for individuals with certain characteristics. To avoid information bias, we excluded individuals that had immigrated (*n*=683,959) or who could not be linked to either their biological mother or father (*n*=12,535). This resulted in a total cohort of 1,839,711 individuals. Data was collected from individuals’ birth until the 31^st^ of December 2013. The resulting age range captured by the study data was 15–35 years.

### Exposure

In Sweden, youth offenders are defined as individuals convicted of a crime on or after their 15^th^ birthday and before their 21^st^ birthday (Eriksson, 2012; SFS 1964:167). Thus, in the present study, the exposure time was between individuals’ 15^th^ and 21^st^ birthdays. We used the NCR to identify and thereby define individuals as *exposed* if they had a conviction on or after their 15^th^ birthday and before their 21^st^ birthday. Individuals were classified as *unexposed* if they did not have a conviction between these ages.

In Sweden, youth offenders are sentenced to community service to a larger extent than adult offenders and youth get shorter imprisonment sentences (Eriksson, 2012; SFS 1964:167). Youth who have been convicted of severe criminal acts such as assault, robbery, murder, rape, or serious drug offences are, just like adults, commonly sanctioned with imprisonment. However, youth offenders that are between 15 and 18 years of age are sentenced to secure youth care instead of prison. Less serious offences are often sanctioned with fines, care or supervision from social services, or community service sentences. In order to examine potential differences within non-imprisoned and imprisoned youth offenders, we obtained information on sanctions from the NCR and further divided exposed individuals into two levels: (1) individuals with a conviction of crime but not an imprisonment sentence, and (2) individuals with a conviction of crime and an imprisonment sentence (secure youth care or prison).

### Outcomes

#### Unintentional injury

Unintentional injury was defined as inpatient admissions and outpatient care for injuries due to unintentional accidents by external causes through the NPR. These injuries are caused by transport accidents (e.g., car accidents) or other external causes for accidents, for example falls, fires, or force of nature (see supplementary 1, Table 1, for ICD-codes; Latvala et al., 2015; Sariaslan, Lichtenstein, Larsson, & Fazel, 2016).

#### Premature death

Premature mortality is usually defined as death before the average life-expectancy in a population, which according to the World Health Organization (WHO) is death before the age of 70 years (World Health Organization, n.d.). In this study, premature death was defined as any death before the age of 35 years, which is the oldest possible age at the end of study (December 31^st^, 2013). Information on time of death and cause of death were obtained from the Cause of Death Register. We also identified specific causes of death using ICD-codes: traffic accidents, non-traffic accidents, suicide, homicide, and non-external causes (see supplementary 1, Table 1, for ICD-codes).

### Follow-up and time-varying exposure

Because youth offenders have a high risk for negative outcomes during adolescence and early adulthood (Aalsma et al., 2016; Farrer et al., 2013), we started the follow-up time from their 15^th^ birthday. By doing this, we allowed individuals to experience any of the outcomes from the start of the exposure time. Because the follow-up time started at the same time as the exposure time (age 15–20), the two levels of exposure were time-varying between individuals’ 15^th^ and 21^st^ birthdays. All individuals were defined as unexposed on their 15^th^ birthday. Individuals remained unexposed from age 15 years until they (1) experienced injury or premature death (outcomes); (2) discontinued follow-up due to emigration or, in the analysis of unintentional injury, died; (3) changed their exposure status before age 21 (i.e., were convicted of a crime before their 21^st^ birthday); or (4) reached the end of the study period (December 31^st^, 2013). We conducted separate analyses for the two outcomes: unintentional injury and premature death. If individuals had experienced an unintentional injury, they still contributed with time in the analyses of premature death.

**Figure 1:**
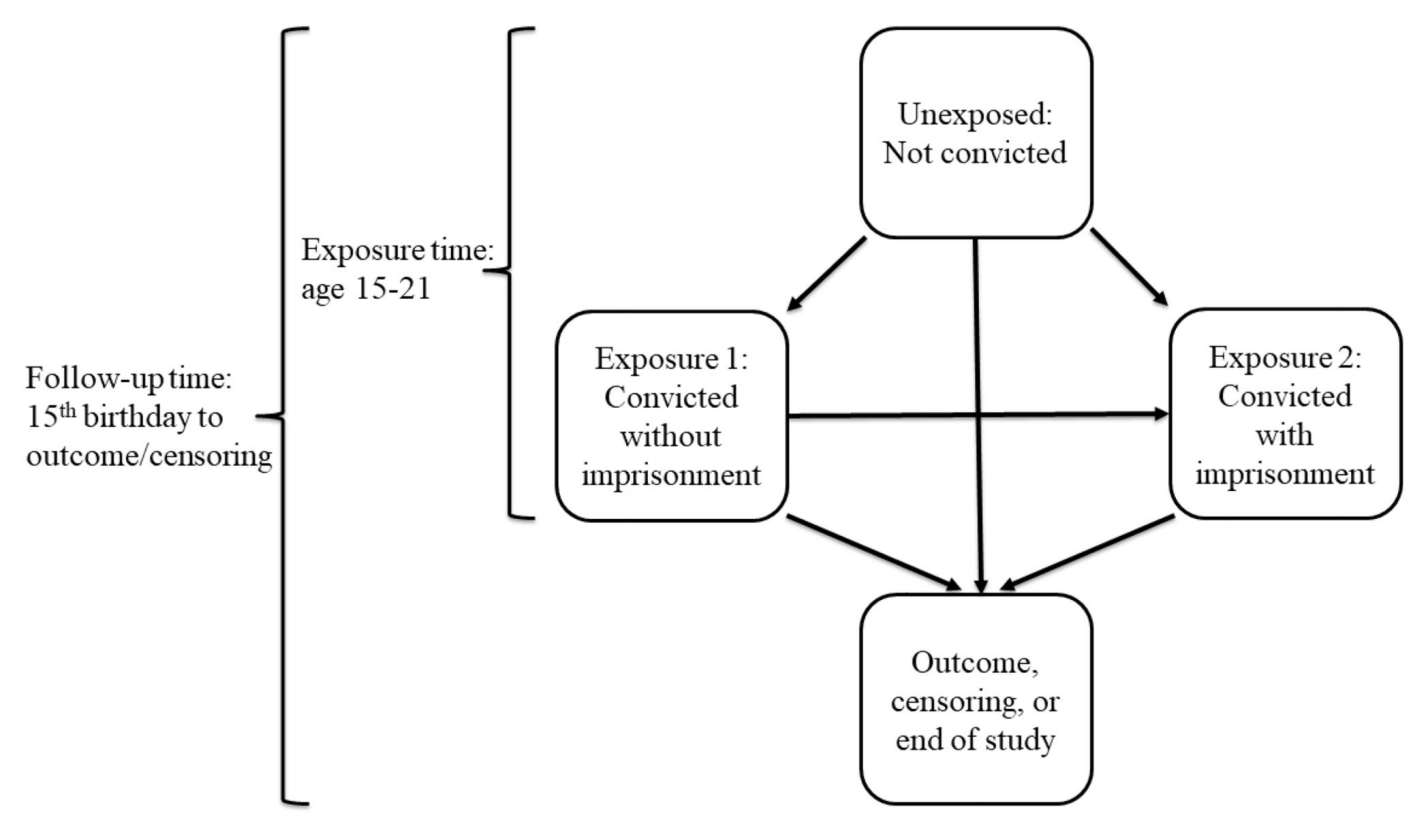
Illustration of time-varying exposure and follow-up time. *Legend*: The same individual could contribute with time in more than one exposure level during the exposure time. Follow-up time began on the same day that the exposure time began. Individuals contributed with time in the exposure level they were in at the end of the exposure time until they experienced the outcome, were censored, or reached the end of the study.

During the exposure time, individuals could experience both exposures of conviction without (level 1) and with (level 2) imprisonment as long as the non-imprisonment sentence occurred first. That is, the time-varying exposure was considered one directional based on the severity of the sentence. Individuals convicted without imprisonment contributed with time in exposure level 1 from the day of conviction until they experienced an outcome, died, emigrated, were convicted with imprisonment (and changed to exposure level 2) or the end of study. Conviction with imprisonment was treated as the most severe exposure level (level 2), so individuals that were first convicted and imprisoned but subsequently convicted without imprisonment did not change to the less severe exposure level. Such an individual would contribute with time only in the most severe exposure level until they experienced one of the outcomes, died, emigrated or the end of study, see Figure 1.

### Risk factors

We examined the role of risk factors in childhood within non-imprisoned and imprisoned youth offenders separately to better understand whether these risk factors exacerbated the risk for unintentional injury and premature death. Information about these risk factors were obtained from individuals’ birth until their 15^th^ birthday (i.e., before the exposure and follow-up time) and were time-invariant.

### Childhood psychiatric disorders

The NPR was used to extract information on *any* psychiatric disorder before the age of 15 years. We further identified specific psychiatric disorders that have an onset in childhood/early adulthood (Sun et al., 2019). These diagnoses were categorized as neurodevelopmental disorders (autism spectrum disorders (ASD), intellectual disability (ID), attention deficit hyperactivity disorder (ADHD), and tic disorders), internalizing disorders (depression and anxiety disorders, including obsessive-compulsive disorder), and externalizing disorders (disruptive behavior disorder (conduct disorder (CD) and oppositional defiant disorder (ODD)). Substance misuse is common among imprisoned youth offenders and about 25% of all youth offenders sentenced to secure youth care have committed a drug-related crime (Pettersson, 2009; The Swedish National Board of Institutional Care, 2021a). Therefore, we included substance use disorders (SUD) in externalizing disorders as well (see supplementary 1, Table 2, for ICD-codes). The reason for analyzing these broader categories instead of separate diagnoses was to increase statistical power.

### Parental criminal convictions

We used the Multi-Generation Register and the NCR to identify whether an index person had a biological mother or father that was convicted of a crime before the index person’s 15^th^ birthday.

### Parental psychiatric disorders

We used the Multi-Generation Register and the NPR to identify whether the index person’s biological mother or father had been diagnosed with *any* psychiatric disorder before the index person’s 15^th^ birthday.

### Covariates

We adjusted for sex since males are more likely to commit crimes and to have a higher risk for unintentional injuries and premature death (Aalsma et al., 2016; Chang et al., 2015; Ekman, Svanström, & Långberg, 2005). We adjusted for birth year to account for cohort effects (Parkinson, Minton, Lewsey, Bouttell, & McCartney, 2017). Lastly, SES has been shown to be associated with both crime and mortality (Galloway & Skardhamar, 2010; Laub & Vaillant, 2000; Trumbetta, Seltzer, Gottesman, & McIntyre, 2010). Since our study population was young, we adjusted for childhood/family SES by using LISA and defined childhood/family SES as either parents’ highest educational level before the index person’s 15^th^ birthday.

### Statistical Analyses

All data management and analyses were performed using SAS software version 9.4 (SAS Institute Inc., Cary, NC) and R 3.6.1 (R Development Core Team, 2020). In the first step of the analyses, we estimated the absolute risk for unintentional injuries and mortality among non-convicted youth, non-imprisoned youth offenders, and imprisoned youth offenders using incidence rates (IR) with 95% confidence intervals (CI).

In the second step, we used Cox proportional hazard regression models to estimate hazard ratios (HR) with 95% CI, with age as an underlying time variable. We used non-convicted youth (unexposed) as the reference group and compared non-imprisoned youth offenders and imprisoned youth offenders (exposure 1 and 2) separately. We treated unintentional injuries and premature death as two separate outcomes. In the first model, we estimated the unadjusted associations between unexposed and the two levels of exposures for the two outcomes. In the second model, we adjusted for sex, birth year, and childhood SES.

In the third step, we examined potential differences in the risk for unintentional injury and premature death within the two exposure levels, non-imprisoned youth offenders and imprisoned youth offenders, separately. The following risk factors were examined: parental criminal convictions, parental psychiatric disorder, any childhood psychiatric disorder, neurodevelopmental disorders, externalizing disorders, and internalizing disorders. We used Cox proportional hazard regression models to estimate HRs and 95% CIs for each risk factor and outcome within non-imprisoned and imprisoned youth offenders separately where individuals without the specific risk factor were used as a reference group. All models were adjusted for sex, birth year, and childhood SES. Due to insufficient statistical power, we excluded neurodevelopmental disorders and internalizing disorders in the analysis of premature death among imprisoned youth offenders.

### Sensitivity analysis

For some individuals, being incarcerated could be a time of a decreased risk for injuries and mortality due to the secure environment and access to health care (Kjelsberg & Laake, 2010). To account for this potential lower risk during the follow-up time for imprisoned youth offenders, we conducted sensitivity analyses in which we compared imprisoned youth offenders to non-convicted youths and separated time imprisoned from time after release from imprisonment. However, in our data, there were no events of injuries or deaths among imprisoned youth offenders during time imprisoned, so we only analyzed time after release from imprisonment. We used Cox proportional hazard regressions with 95% CIs and ran one unadjusted model and a second model adjusted for sex, birth year, and childhood SES for each outcome.

## Results

### Descriptive statistics

Out of the 1,839,711 individuals in the cohort, a total of 208,873 individuals had a conviction of a crime between their 15^th^ and 21^st^ birthday. Table 1 displays information on sociodemographic factors and included risk factors for the total cohort before individuals’ 15^th^ birthday. About 4% (*n*=73,453) of all individuals had a psychiatric diagnosis before age 15. The prevalence for parental criminal convictions was 24%, and 14% of the sample had a parent with a diagnosis of a psychiatric disorder during the index person’s childhood. During the follow-up, 412,034 (22%) individuals experienced an unintentional injury and 8,525 (5%) individuals died. The mean age for unintentional injury was 21.09 years (*SD*=4.62) and the mean age at death was 22.98 years (*SD*=4.70). Among all deaths, 67% were of external causes and 33% were of non-external causes. The most frequent external cause of death was suicide (25% of total deaths), followed by non-traffic accidents (22%), traffic accidents (18%), and homicide (2%).

### Risk for unintentional injury and premature death

The incidence rates for unintentional injury and premature death for unexposed and each level of exposed youth (i.e., non-imprisoned and imprisoned youth offenders) are presented in Table 2. The highest incidence rate for unintentional injury was found among imprisoned youth offenders with 567.59 unintentional injuries per 10,000 person-years, whereas non-convicted individuals had 218.76 injuries per 10,000 person-years. The highest all-cause mortality rate was found among imprisoned youth offenders with 56.36 deaths per 10,000 person-years, whereas non-convicted youth had 3.21 deaths per 10,000 person-years.

Cox proportional hazard models adjusted for sex, birth year, and childhood SES showed that imprisoned youth offenders had the highest risk for both unintentional injury (2.29 [2.19-2.40]) and premature death (10.76 [9.52-12.16]), followed by non-imprisoned youth offenders (1.52 [1.51-1.54], and 3.02 [2.87-3.17], respectively), compared to non-convicted youth (see Table 3).

### Risk factors in childhood in non-imprisoned and imprisoned youth offenders separately

**Figure 2:**
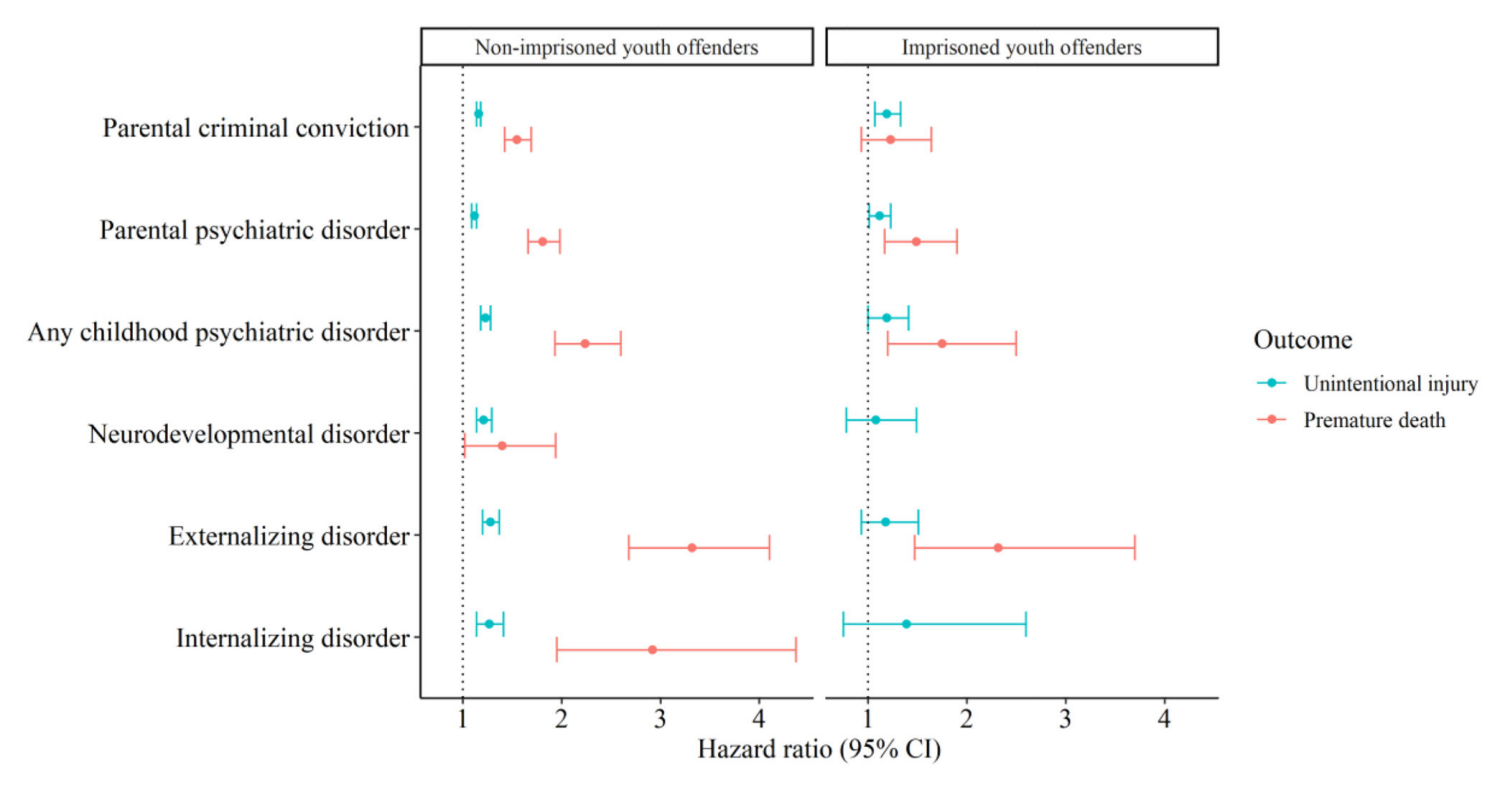
Forest plot with HR and 95% CIs for estimates from analyses for each risk factor, exposure level, and outcome separately, where the reference group for each analysis is individuals without the examined risk factor. *Legend*: Each line in the plot represents one cox regression analysis.

We next analyzed how parental criminal convictions and psychiatric disorders and childhood psychiatric disorders affected the risk of injury and premature death in non-imprisoned and imprisoned youth separately. Results for the risk for unintentional injury and premature death in relation to childhood risk factors in each offender group are presented in Figure 2 (for HR estimates and CIs, see supplementary 2, Table 1).

### Non-imprisoned youth offenders

Among non-imprisoned youth offenders, all risk factors increased the risk for both unintentional injury and premature death. Childhood psychiatric disorders increased the risk the most for both outcomes (HR unintentional injury=1.23 [1.18-1.28]; HR premature death=2.24 [1.93-2.60]), where specifically externalizing disorders posed the highest risk (HR unintentional injury=1.28 [1.20-1.37]; HR premature death=3.32 [2.68-4.10]). Both parental criminal convictions (HR unintentional injury=1.17 [1.15-1.19]; HR premature death=1.54 [1.41-1.68]) and parental psychiatric disorders (HR unintentional injury=1.12 [1.09-1.14]; HR premature death=1.81 [1.66-1.98]) increased the risk for both outcomes. Notably, the estimates were higher for premature death than for unintentional injury.

### Imprisoned youth offenders

Among imprisoned youth offenders, only parental criminal convictions (HR=1.18 [1.06-1.33]) and parental psychiatric disorders (HR=1.12 [1.01-1.23]) increased the risk for unintentional injury, where parental criminal convictions had the highest estimate. For premature death, any parental psychiatric disorder (HR=1.49 [1.17-1.90]), any childhood psychiatric disorder (HR=1.75 [1.20-2.50]), and externalizing disorders (HR=2.32 [1.47-3.70]) increased the risk. No significant increase in risk for premature death was found for parental criminal convictions (neurodevelopmental and internalizing disorders were not included in the analyses due to insufficient power). Similar to non-imprisoned youth offenders, the estimates were higher for premature death than for unintentional injury.

### Sensitivity analysis

Our estimates for the risk of unintentional injuries and premature death for imprisoned youth offenders compared to non-convicted youths were similar when only time after release from imprisonment (i.e., excluding time in imprisonment) was analyzed (for HR estimates and CIs, see supplementary 2, Table 2).

## Discussion

In this longitudinal population-based cohort study, we examined the risk for unintentional injury and premature death among non-imprisoned and imprisoned youth offenders. In line with previous research (e.g., Coffey et al., 2003; Farrer et al., 2013), we found that youth offenders in general had a higher risk to be injured and to die prematurely compared to non-convicted youth. We further extended these findings in three important ways. First, we found that imprisoned youth offenders were at a greater risk of injury and premature death compared to non-imprisoned youth offenders. Second, all childhood risk factors (parental criminal convictions, parental psychiatric disorder, and childhood psychiatric disorders) increased the risk for both unintentional injury and premature death among non-imprisoned youths. Third, although not statistically tested, risk factors seem to play less of a role among imprisoned youth offenders since only a few were significant. In contrast to non-imprisoned youth offenders, only parental criminal convictions and parental psychiatric disorders increased the risk for unintentional injury and only parental psychiatric disorders and own childhood psychiatric disorders increased the risk for premature death. This implies that the need for assessment of risks for these outcomes could vary between non-imprisoned and imprisoned youth offenders.

### Risk for unintentional injury and premature death

In fully adjusted models, our results for unintentional injury showed that imprisoned youth offenders had 2.3 times higher risk than non-convicted youth to experience an unintentional injury. The corresponding estimate for non-imprisoned youth offenders was 1.5. This is in line with previous research showing that youth offenders had around 1.8-3.4 times higher risk to experience an injury than the general youth population (Farrer et al., 2013; Timonen et al., 2003). To our knowledge, this is the first population-based study to examine the risk for unintentional injury in imprisoned *and* non-imprisoned youth offenders. Considering the non-overlapping confidence intervals of the estimates between non-imprisoned and imprisoned youth offenders, our results indicate that imprisoned youth offenders had a higher risk to experience an unintentional injury than non-imprisoned youth offenders.

Similar results were found for premature death, where imprisoned youth offenders had 10 times higher risk of dying before the age of 35 compared to non-convicted youth, and non-imprisoned youth offenders had three times higher risk than non-convicted youth. Again, the non-overlapping confidence intervals for the estimates of non-imprisoned and imprisoned youth offenders suggest that incarceration was related to a higher risk for premature death. Although findings among youth offenders are sparse (Aalsma et al., 2016), this finding is in line with previous work on adults showing that being incarcerated increased the risk for mortality (Daza, Palloni & Jones, 2020; Massoglia, Pare, Schnittker & Gagnon, 2014; Pridemore, 2014; Witteveen, 2022).

The estimates for both unintentional injury and premature death were similar in our sensitivity analysis in which we separated time imprisoned from time after release from imprisonment. This is probably due to that there were no cases of injuries or death for imprisoned youth offenders while they were imprisoned. Further, the change in number of person-years in the sensitivity model is minor due to the short imprisonment sentences among youth offenders.

Our finding that imprisoned youth offenders had a higher risk for both unintentional injury and premature death compared to non-imprisoned youth offenders could be explained by several factors. Incarceration has been shown to be both an acute and a chronic stressor that may negatively affect both mental and general health (see Massoglia & Pridemore, 2015 for a review), which in turn could lead to an increased risk for injuries and mortality after release from imprisonment. In addition, previous research suggested that individuals who engage in more serious criminal offending (e.g., committed violent or multiple crimes) have a more antisocial and riskier lifestyle that puts them at risk for severe adverse outcomes such as injuries or death (Farrer et al., 2013; Kjelsberg & Laake, 2010; Stenbacka, Moberg, & Jokinen, 2019; Zoldre & Fazel, 2012). Since only those who commit a serious criminal offence (e.g., robbery, murder, or rape) receive an imprisonment sentence, incarceration could be a potential indicator for more serious criminal offending or an overall higher risk-profile for other risky behaviors, and in turn explain why the imprisoned youth offenders in the present study had a higher risk for both injuries and premature death compared to non-imprisoned youth offenders.

### The role of childhood risk factors in non-imprisoned and imprisoned youth offenders separately

Further, the present study investigated the role of several risk factors in childhood (i.e., parental criminal conviction, parental psychiatric disorders, and childhood psychiatric disorders) to better understand the association between crime in youth and unintentional injury and premature death in non-imprisoned and imprisoned youth separately. The results showed that among non-imprisoned youth offenders, those who had a parent convicted of a crime, a parent with a psychiatric disorder, or a childhood psychiatric disorder had a higher risk for both unintentional injury and premature death compared to those without these risk factors. Among imprisoned youth offenders, only parental criminal conviction and parental psychiatric disorder increased the risk for unintentional injury while parental psychiatric disorder and childhood psychiatric disorder increased the risk for premature death.

That all risk factors were significant among non-imprisoned youth offenders, but only a few were significant among imprisoned youth offenders implies that the risks for unintentional injury and premature death varied more among non-imprisoned youth offenders while the risks were high among most imprisoned youth offenders. Consequently, our findings suggest that intervention or preventative efforts aimed at youth offenders with the highest risk for unintentional injury or death should target all imprisoned youth offenders. In other words, additional risk assessment may not be needed among these youth. Risk assessment could instead be useful among non-imprisoned youth offenders to identify those with childhood risk factors and who could thus be targeted for intervention efforts.

In both youth offender groups, childhood psychiatric disorders increased the risk for both unintentional injury and premature death. Previous research showed that adult offenders with a psychiatric disorder had an increased risk for injuries and mortality (Timonen et al., 2003; Zoldre & Fazel, 2012), therefore our findings add to the literature by showing that psychiatric disorders played a significant role for these outcomes across the lifespan (i.e., also in childhood).

Externalizing disorders (i.e., disruptive behavior disorders and substance use disorders) had the highest estimates for premature death across all risk factors and offender groups. This is in line with a previous population-based study on adult offenders released from prison showing that substance use was the only significant risk factor for mortality when controlling for other psychiatric disorders and related confounders (Chang et al., 2015). Combined, these results indicate that substance use is an important risk factor to consider not only in adults, but also among youth offenders. In addition, in the present study, we only obtained information on substance misuse before individuals’ 15^th^ birthday and hence before any criminal convictions. It is well known that substance misuse and criminal offending are strongly linked (Pierce et al., 2017), therefore youth offenders may be likely to also develop substance misuse. This supports the notion of the benefits of substance use treatment to prevent injuries and death.

Parental criminal conviction increased the risk for unintentional injury and premature death in non-imprisoned youth offenders, while it only increased the risk for unintentional injuries, and not premature death, among imprisoned youth offenders. Parental psychiatric disorders increased the risk for both outcomes in both offender groups. Previous research considering family history as a risk factor for mortality among youth offenders is sparse, but our results are in line with a population-based study showing that family history of crime, psychiatric disorders, and substance use increased the risk for suicide among adolescent violent offenders (Björkenstam et al., 2018). Although we did not examine the role of family history of criminal conviction and psychiatric disorders in the risk for criminality in youth, we do know from previous research that both criminality (Frisell et al., 2011), psychiatric disorders (Pettersson et al., 2019), and mortality (Oyen et al., 2009) aggregates in families. This implies that the associations between childhood risk factors, criminality in youth, and later injuries or death could be a result of both genetic and environmental factors working in tandem.

Shared genetic vulnerability to psychiatric disorders has been shown to increase the risk for both criminal behavior, suicide attempts and injuries due to accidents (Fazel & Runeson, 2020; Mok et al., 2016). Thus, youth who have a parent with a psychiatric disorder have a higher risk to develop a psychiatric disorder themselves, which in turn could increase the risk for injuries and death. Further, they also have a higher risk to engage in criminal behavior, which entails an additional effect on the risk for these outcomes. Moreover, youth with a parent that have been convicted of a crime or have a psychiatric disorder are at risk for being exposed to early negative environmental factors such as violence, neglect, harsh parenting styles, or financial problems (Mok et al., 2016). These environmental factors in combination with a genetic vulnerability could increase the risk of impulsive, aggressive, or antisocial behaviors. These behaviors may in turn increase the risk of individuals to engage in criminal and other risky behaviors that could lead to injuries or death.

### Limitations

There are three main limitations that need to be considered when interpreting the results in the present study. First, we used data from inpatient or outpatient specialist care to identify individuals with psychiatric disorders. Since only the more severe cases are admitted to specialist care not all cases with disorders are covered in the data. Therefore, the prevalence of psychiatric disorders for both youth offenders and for their parents was likely underestimated. Also, not all criminal acts lead to conviction and offenders tend to not seek medical care to the same extent as non-offenders (Howerton et al., 2007). Thus, the prevalence of criminal offences and unintentional injuries are also likely to be underestimated. Additionally, the incomplete coverage of psychiatric disorders in combination with a rare outcome such as death before age 35 years led to insufficient power in our analyses of psychiatric disorders and premature death among imprisoned youth offenders, hence increasing the risk for type II errors. Given the wider confidence intervals in the analyses with imprisoned youths only (see Figure 2), our results within this group should be interpreted cautiously.

Second, the use of large datasets made it possible to adjust for several important confounders (i.e., SES, sex, and age). The included risk factors and confounders seemed appropriate based on previous studies (Aalsma et al., 2016; Chang et al., 2015; Massoglia & Pridemore, 2015; Pettersson, 2009; Salias et al., 2006). Nevertheless, other potentially important confounders such as environmental factors (e.g., antisocial peers) or personality traits (e.g., impulsivity or self-control) are not covered in the Swedish registers and therefore could not be included in this study. Future studies should include important confounders to avoid risk for confounding associations. The present study also did not examine potential mediators between criminal convictions and unintentional injuries or premature death such as poor mental and general health. Factors such as these could be the result of a criminal lifestyle and in turn lead to unintentional injury or premature death. In addition, we did not consider later criminal convictions that occurred after each individual’s 21^st^ birthday. Most criminal offending occurs during adolescence and early adulthood (Loeber & Farrington, 2014; Quetelet, 2003), suggesting that a large portion of adult criminals have also been convicted in youth. However, there could be individuals that are convicted of a crime for the first time later in life and thereby also have a higher risk for these outcomes. Thus, adult criminal convictions could be a potential mediator that was not included in this study.

Third and last, we used data from Swedish national registers. Due to differences in health care policies and criminal justice systems, the results may not be generalizable to other countries with less comprehensive welfare systems and more punitive criminal justice systems. Future research will need to investigate the role of different systems in unintentional injury and premature death. We also excluded individuals that had immigrated to Sweden to avoid information bias of factors during childhood. In Sweden, and in the Nordic countries in general, individuals with an immigrant background are overrepresented in the offender population (The Swedish National Council for Crime Prevention, 2021b). Immigrants can also differ on several factors from non-immigrants (e.g., SES, psychiatric disorders, general health), so our results should not be generalized to immigrant populations.

### Implications and conclusions

The present study has some implications for future research. Given that the registers likely only captured instances of severe psychiatric illness, which were relatively few in number, we did not have sufficient power to examine specific diagnoses among youth offenders. Previous research showed that some disorders increased the risk for death more so than other disorders (e.g., substance use, see Chang et al., 2015). This indicates that future studies should use large samples and a more sensitive dimensional approach to symptoms of psychiatric disorders to examine the role of specific diagnoses separately. It would also be of interest to examine additive effects of risk factors and to have a longer follow-up period to elucidate the role of risk factors in different stages of life in the risk for unintentional injuries and death.

In the present study, we were not able to examine the risk of cause-specific deaths due to insufficient power. Thus, future studies should employ cause-specific analyses to examine the role of risk factors for different external causes of death among youth offenders. However, most deaths in this study were due to external causes and 25% of all deaths were due to suicide. Interventions should therefore be aimed at suicide prevention among youth offenders.

Lastly, our study showed robust risk factors for injuries and death among youth offenders. Importantly, these risk factors are modifiable and could be targeted by interventions. For example, therapeutic interventions could reduce the symptom burden of mental disorders, or family-based interventions such as functional family therapy or parent management training could help to prevent criminal behavior in youth (Welsh & Farrington, 2006). However, the effect of intervention efforts among youth offenders to prevent injuries and death needs to be further researched (Welsh, Zane, & Reeves, 2021). Our results also suggest that research on prediction models for injuries and death among youth offenders is a necessary next step (see for example Fazel, Wolf, Larsson, Mallet, & Fanshawe, 2019; Yu et al., 2022). This could have implications for risk assessment among youth offenders to better guide intervention efforts.

To conclude, our study showed that youth offenders overall had a higher risk for both unintentional injuries and premature death compared to the general youth population. sFurthermore, imprisoned youth offenders had a higher risk for these outcomes than non-imprisoned youth offenders. This study also found robust modifiable childhood risk factors for injury and mortality within non-imprisoned and imprisoned youth offenders separately. Risk factors in childhood could be used to identify youth offenders with the highest risk for these outcomes. However, these risk factors seemed to be less important in assessing risk *among* imprisoned youth offenders because this seemed to be a more homogenous group of individuals who were all at high risk. Therefore, it may be more efficient to target all imprisoned youth offenders in interventions. Although the included risk factors in this study are general and frequent among youth offenders, and more research on detailed specific risk factors is needed, our results indicates that risk assessments for unintentional injuries and premature death could be beneficial among non-imprisoned youth offenders where those with highest risk could be targeted for interventions. Nevertheless, this study showed that risk factors in childhood increased the risk for these outcomes and therefore remain important to consider in the association between criminality in youth and later unintentional injuries and premature death.

## Supplementary Material

Supplementary Table

## Figures and Tables

**Table 1:** Descriptive statistics for the total population before their 15^th^ birthday.

		Total population	
		N	%
Sex	Male	947,820	52
	Female	891,891	48
Parent education (i.e., Childhood SES)	≤9 years	121,044	6
	10-12 years	913,257	50
	13≤ years	804,353	44
Parental criminal convictions	No	1,010,955	55
	Yes	444,586	24
Parental psychiatric disorder	No	1,578,991	86
	Yes	260,720	14
Any psychiatric disorder in childhood	No	1,766,258	96
	Yes	73,453	4
Neurodevelopmental disorders	No	1,813,244	99
	Yes	26,467	1
Internalizing disorders	No	1,829,732	99
	Yes	9,979	1
Externalizing disorders	No	1,830,013	99
	Yes	9,698	1
Death between age 15–35	No	1,831,186	99
	Yes	8,525	1
Unintentional injury between age 15-35	No	1,427,677	78
	Yes	412,034	22

**Table 2:** Incidence rate per 10,000 person-years for unintentional injury and premature death.

	N at risk	N having event	Person years	IR per 10,000 person-years [95% CI]
**Unintentional Injury**				
Non-convicted youth	1,839,711	358,017	16,365,692	218.76 [218.04-219.48]
Non-imprisoned youth offenders	190,324	52,169	1,525,977	341.87 [338.95-344.81]
Imprisoned youth offenders	4,820	1,848	32,558	567.59 [542.12-593.88]
**Premature death**				
Non-convicted youth	1,839,711	5,867	18,293,226	3.21 [3.13-3.29]
Non-imprisoned youth offenders	207,647	2,376	1,910,360	12.44 [11.94-12.94]
Imprisoned youth offenders	6,098	282	50,032	56.36 [50.04-63.20]

**Table 3:** HR with 95% CI from Cox proportional hazard regression models for non-imprisoned and imprisoned youth offenders where non-convicted youth are the reference group.

	Unadjusted HR [95% CI]	Adjusted for sex, birth year, and childhood SES HR [95% CI]
**Unintentional injury**		
Non-convicted youth (n=1,839,711)	Reference	Reference
Non-imprisoned youth offenders (n=190,324)	1.63 [1.61-1.64]	1.52 [1.51-1.54]
Imprisoned youth offenders (n=4,820)	2.75 [2.63-2.88]	2.29 [2.19-2.40]
**Premature death**		
Non-convicted youth (n=1,839,711)	Reference	Reference
Non-imprisoned youth offenders (n=207,647)	3.55 [3.39-3.73]	3.02 [2.87-3.17]
Imprisoned youth offenders (n=6,098)	15.10 [13.39-17.04]	10.76 [9.52-12.16]
