# Supplementary material for: A Population-Based Study of Unintentional Injury and Premature Death among Non-imprisoned and Imprisoned Youth Offenders
Source: J Crim Justice. Author manuscript; Available in PMC 2025 Sep 2. (PMC7618064; doi:10.1016/j.jcrimjus.2022.102009)
Supplement: Supplementary Table [file EMS208262-supplement-Supplementary_Table.docx]

**Supplementary**

Table S1. *Swedish ICD-codes for unintentional innjury and premature death.*

| **Outcome** | **Cause** | **ICD-8** | **ICD-9** | **ICD-10** |
| --- | --- | --- | --- | --- |
| Unintentional injury | Unintentional injury | E800-E929 | E800-E869, E880- E928 | V01-X59 |
| Premature death | Traffic accident | E807-E846 | E807-E849 | V01-V99, Y85-Y89 |
|  | Non-traffic accident | E859-E929 | E859-E866 | W00-W99, X00-X59, Y10-Y34 |
|  | Suicide | E950-E959 | E950-E959 | X60-X84 |
|  | Homicide | E960-E969 | E960-E969 | X85-X99, Y00-Y99 |
|  | Non-external | 000-796.99 | 001-799 | A00-R99 |

Table S2. *Swedish ICD-codes for psychiatric disorders.*

| **Psychiatric diagnosis** | **ICD-8** | **ICD-9** | **ICD-10** |
| --- | --- | --- | --- |
| ASD | 295.80, 299.99 | 299 | F84 |
| ID | 310-315 | 317–319 | F70-F73, F78-F79 |
| ADHD | Not applicable | 314 | F90 |
| Tic | 306.2 | 307C | F95 |
| Disruptive behavior disorder (CD/ODD) | 308.99 | 312, 314W-X | F90.1, F91 |
| Depression | 296.2, 300.4 | 296D, 300E, 311 | F32- F34 |
| Anxiety disorder | 300 (excluding 300.4) | 298B, 300 | F40-42 |
| SUD | 291, 294.1, 303–304 | 291–292, 303–305 | F10-F19 |

Table S3. *HR and 95% CIs from analyses for each risk factor, exposure level, and outcome separately. Individuals that did not have the risk factor within each stratification were used as a reference group.*

| **Outcome** | **Exposure level** | **Risk factor** | **HR** | **Lower CI** | **Upper CI** |
| --- | --- | --- | --- | --- | --- |
| Unintentional injury | Non-imprisoned youth offenders | Parental psychiatric disorder | 1.12 | 1.09 | 1.14 |
|  |  | Family history of criminal convictions | 1.16 | 1.14 | 1.18 |
|  |  | Any childhood psychiatric disorder | 1.23 | 1.18 | 1.28 |
|  |  | Neurodevelopmental disorders | 1.21 | 1.14 | 1.29 |
|  |  | Externalizing disorders | 1.28 | 1.20 | 1.37 |
|  |  | Internalizing disorders | 1.27 | 1.14 | 1.41 |
|  | Imprisoned youth offenders | Parental psychiatric disorder | 1.12 | 1.01 | 1.23 |
|  |  | Family history of criminal convictions | 1.19 | 1.07 | 1.33 |
|  |  | Any childhood psychiatric disorder | 1.19 | 1.00 | 1.41 |
|  |  | Neurodevelopmental disorders | 1.08 | .78 | 1.49 |
|  |  | Externalizing disorders | 1.18 | .93 | 1.51 |
|  |  | Internalizing disorders | 1.39 | .75 | 2.60 |
| Premature death | Non-imprisoned youth offenders | Parental psychiatric disorder | 1.81 | 1.66 | 1.98 |
|  |  | Family history of criminal convictions | 1.55 | 1.42 | 1.69 |
|  |  | Any childhood psychiatric disorder | 2.24 | 1.93 | 2.60 |
|  |  | Neurodevelopmental disorders | 1.40 | 1.02 | 1.94 |
|  |  | Externalizing disorders | 3.32 | 2.68 | 4.10 |
|  |  | Internalizing disorders | 2.92 | 1.95 | 4.37 |
|  | Imprisoned youth offenders | Parental psychiatric disorder | 1.49 | 1.17 | 1.90 |
|  |  | Family history of criminal convictions | 1.23 | .93 | 1.64 |
|  |  | Any childhood psychiatric disorder | 1.75 | 1.20 | 2.50 |
|  |  | Neurodevelopmental disorders | na | na | na |
|  |  | Externalizing disorders | 2.32 | 1.47 | 3.70 |
|  |  | Internalizing disorders | na | na | na |

Table S4. *HR with 95% CI from Cox proportional hazard regression models for imprisoned youth offenders after release from imprisonment where non-convicted youth are the reference group.*

|  | **Unadjusted**  **HR [95% CI]** | **Adjusted for sex, birth year, and childhood SES**  **HR [95% CI]** |
| --- | --- | --- |
| **Unintentional injury** | | |
| Non-convicted youth  (n=1,839,711) | Reference | Reference |
| Imprisoned youth offenders  (n=4,820) | 2.78 [2.65-2.92] | 2.33 [2.22-2.44] |
| **Premature death** | | |
| Non-convicted youth  (n=1,839,711) | Reference | Reference |
| Imprisoned youth offenders  (n=6,098) | 15.74 [13.92-17.80] | 11.29 [9.97-12.80] |
